# Continuous and controllable electro-fabrication of antimicrobial copper-alginate dressing for infected wounds treatment

**DOI:** 10.1007/s10856-021-06619-2

**Published:** 2021-11-24

**Authors:** Shijia Wang, Xiaoli Liu, Miao Lei, Junjie Sun, Xue Qu, Changsheng Liu

**Affiliations:** Key Laboratory for Ultrafine Materials of Ministry of Education, Frontiers Science Center for Materiobiology and Dynamic Chemistry, School of material science and engineering, East China University of Science and Technology, Shanghai, 200237 China

## Abstract

The contamination of chronic wound with bacteria especially methicillin-resistant *Staphylococcus aureus* (MRSA) is considered as the major factor interferencing normal wound healing. There still remain great challenges in developing safe and effective wound dressings with wide-spectrum antibacterial functions. Alginate hydrogel is a common dressing for wound treatment. Copper is one of the trace elements in human body with inherent antibacterial activity. Traditional methods for preparing a structure-controlled copper-alginate antibacterial matrix are difficult however, due to the fast and uncontrolled gelation between alginate and metal ions. In this work, we report an electrodeposition method for rapid fabrication of copper cross-linked alginate antibacterial films (Cu^2+^-Alg) with controlled structure and copper content, which is relied on an electrical signal controlled release of copper ions from the reaction of insoluble salt Cu_2_(OH)_2_CO_3_ and the generated protons via water electrolysis on anode. The results prove that the physical structure and chemical composition of the electrodeposited Cu^2+^-Alg films can be continuously modulated by the imposed charges during electrodeposition. In vitro tests demonstrate the film has Cu^2+^ content-dependent bactericidal activities. Film’s cytocompatibility is well controlled by the imposed charges for Cu^2+^-Alg fabrication. The MRSA infected wound model in vivo also indicates that Cu^2+^-Alg film can effectively eliminate bacterial infection and suppress host inflammatory responses. We believe this study demonstrates a convenient and controllable strategy to fabricate alginate antibacterial dressings with potential applications for infected wound treatment. More broadly, our work reveals electrodeposition is a general and simple platform to design alginate films with versatile functions.

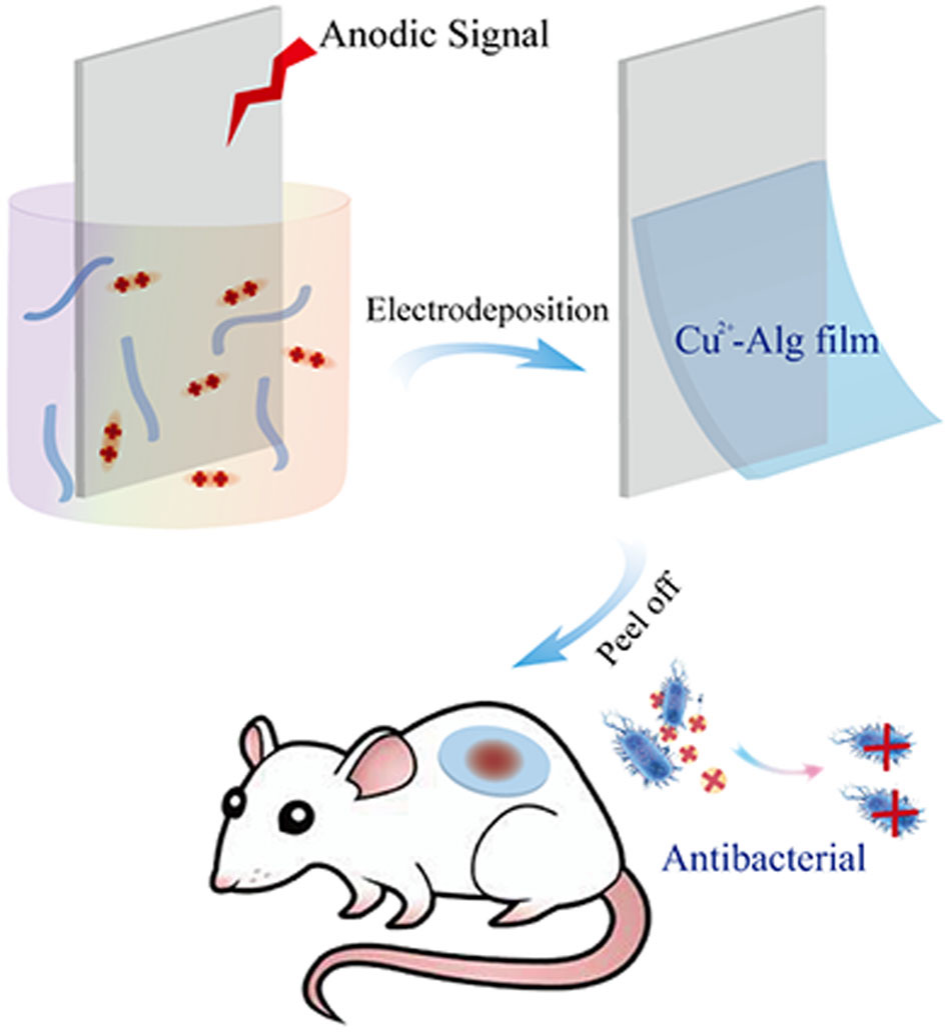

## Introduction

As the aging population and diseases such as diabetes, obesity, and heart disease are proven to be associaDted with wound healing, the risk of tractable wounds being transferred to chronic wounds increases dramatically [1, 2]. Normally, the natural process of wound healing involves four classic stages: hemostasis, inflammation, proliferation, and remodeling [3, 4]. Chronic wounds are characterized by prolonged inflammation and delayed healing, which are associated with diminished neovascularization and re-epithelization [5]. Bacterial infections are considered to be a major local factor to the detriment of wound healing, which lead to a prolonged excessive inflammatory response and delay in collagen synthesis and epithelialization [6, 7]. In particular, microbiological studies have confirmed that chronic wound infection with methicillin-resistant *Staphylococcus aureus* (MRSA) is a growing worldwide problem [8–10]. Hence, superior wound treatment using dressings with wide-spectrum antimicrobial bioactivities is highly recommended [11, 12].

Alginate (Alg) is a commonly used dressing material due to its advantages such as readily available, biocompatible and non-toxic [13–16]. Its organic part is composed of M units (β-D-mannuronate) and G units (α-L-guluronate) and these two units are linked by 1,4 glycoside bonds in different ratios [17]. Most polyvalent metal ions (such as Cu^2+^, Ca^2+^, Zn^2+^, Co^2+^, Ba^2+^) can undergo an ion exchange reaction with an aqueous sodium alginate solution to form another insoluble alginate [18, 19]. And this process can cause a so-called “egg-box” structure with a planar two-dimensional manner, which further leads to the formation of ionic cross-linked hydrogels [20]. Copper is one of the 26 essential nutrients in human, participating in the metabolic activities [21]. Additionally, copper has been widely reported for its broad antibacterial properties [22–25]. In 2008, copper was the first metal to receive EPA certification [26]. The report pointed out that elemental copper, copper oxide, copper ions (Cu^2+^), etc., all have excellent antibacterial properties against different strains [27]. However, Cu has potential hazards in excess of cellular needs, so the delivery of Cu has to be strictly controlled in a narrow therapeutic window and precisely regulated for potential biomedical applications [28–30].

So far, Cu^2+^-Alg matrix was mainly investigated with respect to its antimicrobial activity [31], while few reports regarded it as a potential candidate for wound dressings. This is mainly because that the conventional methods for preparing Cu^2+^-Alg based materials, like impregnation and electrostatic extrusion, are time-consuming and labor-intensive [32, 33]. Methods like impregnation also have difficulties in obtaining homogeneous films with accurate control of Cu^2+^ contents due to the strong affinity between Cu^2+^ and Alg [34, 35]. Therefore, exploring a simple, mild and reproducible method for preparing highly uniform Cu^2+^-Alg dressings and accurately controlling the copper content is highly required for developing advanced antibacterial dressings.

Electrodeposition technology has attracted a lot of attention in material fabrication field in recent years [36–38]. Mechanically, programmable electric signals with exquisite spatiotemporal and quantitative control are imposed to initiate varying electrolytic reactions, which can induce material formation and further modification in-situ [39–42]. Its main advantages are including (1) simple, mild and rapid material molding [16, 43]; (2) continuous and controllable modulation of material’s inner structures and chemical characteristics [39, 44]; (3) creation of complex 3D structures by changing the shape of electrodes [45–47].

**Scheme 1:**
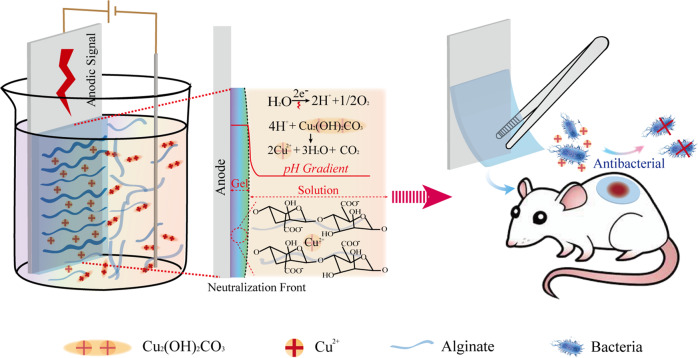
Mechanism of copper alginate (Cu^2+^-Alg) film prepared by electrodeposition: electrochemically generated protons from the anode surface trigger the release of Cu^2+^ ions from insoluble Cu_*2*_(OH)_*2*_CO_3_ particles. The free Cu^2+^ ions interact with the alginate and cross-link the polymer chains to form a hydrogel film on the electrode surface rapidly. The peeled Cu^2+^-Alg dressing releases dose-controlled Cu^2+^ to fight bacteria and improve infected wound healing

Here, we propose a novel electro-fabrication methodology to controllably prepare Cu^2+^-Alg dressings. Scheme 1 summarizes the mechanism for Cu^2+^-Alg fabrication and its potential application as antibacterial dressing. Cu_2_(OH)_2_CO_3_ serves as the resource of copper ions. After suspending Cu_2_(OH)_2_CO_3_ into the Alg solution, a three-electrode system is placed into it and an instantaneous current is applied to trigger the electrolysis of water. Protons are thus continually generated to form a pH gradient at the anode surface, by which the suspended Cu_2_(OH)_2_CO_3_ particles near the anode area are gradually dissolved to release Cu^2+^ to crosslink alginate. During this process, a uniform Cu^2+^-Alg hydrogel is formed on the anodic surface, which can be peeled off completely and serve as a free-standing film. This electro-fabrication method offers several unique advantages over other conventional methods: (1) films can be prepared in a quite short time (within a few minutes); (2) Cu^2+^ distribution is more uniform due to a controlled ion releasing mechanism; (3) the physical structure (i.e. thickness) and chemical composition (i.e. Cu content) of the films can be precisely modulated via charge transfer (Q); (4) porous structure is created by the simultaneously generated O_2_ and CO_2_ on anode. We demonstrate the Cu^2+^-Alg film confers copper ion content-dependent antimicrobial activity in vitro. Its onset time is shorter than commercial silver dressings, while has comparable or better cytocompatibility decided by the Cu^2+^ amount. A rat subcutaneous antimicrobial model demonstrates that the electro-fabricated Cu^2+^-Alg film has strong antibacterial ability towards MRSA in vivo. We believe our work provides a simple and controllable novel method for fabricating Cu^2+^-Alg based antibacterial hydrogel dressing for chronic wound management. More broadly, this study demonstrates the electrodeposition method is promising to construct new structure and confer functions for achieving high performance materials.

## Materials and methods

### Materials

Sodium alginate and cupric subcarbonate were purchased from Sigma-Aldrich. Live/dead BacLight Bacterial Viability Kit was purchased from Invitrogen. The commercial dressing Biatain^®^ Alginate Ag was purchased from Advanced Medical Solutions Ltd. Other reagents were purchased form Aladdin chemistry unless otherwise specified. The chemicals were used without further purification.

### Electro-fabrication of Cu^2+^/Ca^2+^-Alg films

The three-electrode system (CHI660E, Chenhua Shanghai) was used for electrodeposition, employing platinum plate (1.5 × 1.5 cm^2^) as the working electrode (anode), Pt wire as the counter electrode (cathode) and Ag/AgCl as the reference electrode.

For Cu^2+^-Alg films, the electrolyte was prepared by dissolving sodium alginate (0.8 wt%) and Cu_2_(OH)_2_CO_3_ (0.5 wt%) in ultrapure water with constant stirring for 5 h. The electrodeposition process was performed by immersing the electrodes into electrolyte and then applying a constant current density (1.78–4.44 mA/cm^2^ on demand) for predetermined time (90 s). The resultant copper alginate-coated electrode was rinsed extensively with ultrapure water. After then, a free-standing Cu^2+^-Alg film was peeled off from the anode.

The consumed anodic charges were calculated according to the Eq. (1):

1 $${{{{{\mathrm{Anodic}}}}}}\,{{{{{\mathrm{charge}}}}}}\,\left( {{{{{\mathrm{Q}}}}}} \right){{{{{\mathrm{ = }}}}}}{\int} {i\,dt;\,{{{{{\mathrm{C}}}}}}}$$

where i is the instant anodic current density (A/cm^2^), t is the anodic oxidation time (sec).

Cu^2+^-Alg films prepared by varying charges are denoted as (Cu^2+^-Alg)_q_, where q indicates the consumed charge amount.

According to our previously reported method [16], the Ca^2+^-Alg film was prepared by dissolving sodium alginate (1 wt%) and CaCO_3_ (0.5 wt%) in ultrapure water and employing electrodeposition method as same as mentioned above.

### Characterizations of Cu^2+^-Alg films

The hydrogel films’ thickness was tested by an electronic spiral micrometer.

The hydrogel films were first frozen at −18 °C for 12 h in the freezer (KK20V40TI, Siemens, Germany) and then freeze-dried at a temperature under 30 °C and a pressure of 18 Pa for 24 h in a freeze-dryer (SCIENTZ-10N, Ningbo Scientz Biotechnology Co, Ltd., China).

The freeze-dried films were carefully handled and cut to extract the fraction of interest. SEM morphological analysis was performed on the scanning electron microscope (S-4800, Hitachi) at an accelerating voltage of 15 kV. The obtained SEM surface images were analyzed by Image J to get the pore size. Meanwhile, the fracture surface of Cu^2+^-Alg films was also be observed by SEM and the obtained images were employed to measure the thickness of freeze-dried films. The elemental analysis and compositional distribution of Cu^2+^-Alg films were measured using energy dispersive spectroscopy (QUANTAX 400-30, BRUKER AXS).

The interconnected porosity was calculated as the interconnected void volume over the total volume. Experimentally, to determine the total volume, films were soaked in ethanol for 1 h and weighted. A Kimwipe was then used to wick away ethanol within interconnected pores, and the films were weighted once again. The interconnected void volume was calculated as the volume of ethanol wicked from the films [39, 48].

To evaluate the films’ mechanical properties, a tensile test was carried out by an ElectroForce^®^ test instrument (Load Frame 3230 System, BOSE, USA) at stretch velocity of 0.01 mm/s. The dry films were cut into strips (5 mm in width with an initial length of 10 mm). The stress and strain at failure of different films were obtained from the stress strain curve. (*n* = 3).

### Quantitative analysis of Cu^2+^ content

Electrodeposited Cu^2+^-Alg films with a series of charge amount were dissolved in an ion chelator, sodium citrate solution (2 mg/mL), respectively. Then it was placed in a 37 °C constant temperature oscillation box to fully dissolve films and free the Cu^2+^ cross-linked within the alginate films. Followed by centrifuging (8000 r/min, 10 min), the supernatant was analyzed by an inductively coupled plasma atomic emission spectrometer (ICP-AES, Perkin-Elmer Optima). All tests were performed in triplicate.

### Decomposing of Cu^2+^-Alg film

A (Cu^2+^-Alg)_0.54C_ film with the size of 1.5 × 1.5 cm^2^ was put into 1 mL phosphate buffer saline (PBS) media and then placed in the 37 °C shaker. The decomposing time of (Cu^2+^-Alg)_0.54C_ film was recorded.

### In vitro antimicrobial experiments

#### Bacterial culture and preparation

Gram-negative bacteria *Escherichia coli* (ATCC 25922), gram-positive bacteria *S. aureus* (ATCC 25923) and MRSA acquired from hospital (Second Affiliated Hospital of Zhejiang University) were used in this study. Both gram-negative and gram-positive bacteria were firstly grown in the corresponding media, Luria-Bertani and Tryptic Soy Broth, respectively, for 24 h in a 37 °C oscillation box. After quantified by diluted plate assay, the bacterial suspensions were washed completely to remove the bacterial culture media and re-suspended by 0.9% physiological saline to a certain concentration, following stored in 4 °C for further use.

#### Antibacterial test

*S. aureus* and *E. coli* were chosen in this study. Electrodeposited copper alginate film (Cu^2+^-Alg)_0.54C_ (1.5 × 1.5 cm^2^) was put into the 24 well plate for test. The bacterial culture plate was set as the negative control sample and the commercial dressing Biatain^®^ Alginate Ag (1.5 × 1.5 cm^2^) was set as positive control sample. For each group, 1 mL of bacteria solution (10^7^ CFU/mL) was added into the sample well, and incubated at 37 °C. Each group had three replicate samples.

After incubation for 0.5 and 10 h, the number of bacteria was obtained by using dilution plate counting. The antibacterial efficiency was calculated by the following formula:

2 $${{{\mathrm{Antibacterial}}}}\,{{{\mathrm{efficiency}}}}\,\left( \% \right) = \left[ {\left( {{{{\mathrm{A}}}} - {{{\mathrm{B}}}}} \right)/{{{\mathrm{A}}}}} \right] \times 100\%$$

where A is the number of bacterial colonies on blank control, B is the number of bacterial colonies on films.

#### Comparison of antibacterial properties within different films

In order to evaluate and compare the antibacterial effect of electrodeposition Cu^2+^-Alg films prepared under different conditions, three groups (Cu^2+^-Alg)_0.36C_, (Cu^2+^-Alg)_0.54C_, (Cu^2+^-Alg)_0.72C_ were used in this experiment. All the groups were placed into the 24 well plate for test with the same size of 1.5 × 1.5 cm^2^. The bacterial culture plate was set as the negative control sample. Each group has three replicate samples. Then, 1 mL of *E. coli* suspensions (10^7^ CFU/mL) was added into each sample well, and incubated at 37 °C for 10 h. After that, the killing efficiency of *E. coli* was calculated by determined dilution for plate counting as mentioned before.

#### The inhibition zone assay

(Cu^2+^-Alg)_0.54C_ film was cut into 8 mm diameter discs for test. Electrodeposited calcium alginate film (Ca^2+^-Alg)_0.54C_ was used as the negative control sample with the same size. 200 μL of *S. aureus* and *E. coli* suspensions (10^7^ CFU/mL) were firstly introduced onto the agar plates, and then the films were placed onto the plates and incubated for 12 h at 37 °C.

#### The protein leakage assay

(Cu^2+^-Alg)_0.54C_ films were co-incubated with *S. aureus* and *E. coli* suspensions in the 24 well plate and the blank plate was set as the negative control sample. The volume and concentration of bacteria suspensions for each well was 1 mL and 1 × 10^7^ CFU/mL, respectively. After 10 h incubation, the bacteria suspensions were collected through centrifuging with 8000 rpm for 5 min. Finally, the protein concentration in the supernatant was determined by a BCA protein kit (BCA assay, beyuntime).

#### SEM observation of bacterial morphology

The bacteria before and after (Cu^2+^-Alg)_0.54C_ film treatment for 10 h were fixed in 2% glutaraldehyde (GA) for 2 h. Then the immobilized bacteria were collected by centrifugation at 8000 rpm for 10 min, and then experienced gradient dehydration. Afterwards, the bacteria solution was dropped onto silicon wafer, followed by freeze-drying and SEM imaging.

#### Live/dead bacterial assay

Live/dead bacterial assay was performed to examine the viability of bacteria before and after (Cu^2+^-Alg)_0.54C_ films treatment. For each group, 1 mL of bacteria solution (10^7^ CFU/mL) was added onto the plate with film, and then incubated for 10 h. The bacteria solution was mixed with 100 μL of dye solution containing 1.67 mM of SYTO 9 dye and 20 mM of propidium iodide for 20 min at room temperature. Then the bacteria were imaged using a confocal laser scanning microscopy.

### Biocompatibility evaluation

#### Cell culture and preparation

Human umbilical vein endothelial cells (HUVECs) were chosen to estimate the biocompatibility of Cu^2+^-Alg films and Ag dressing, firstly grown in Dulbecco’s modified Eagle’s medium and then cultured in tissue culture flasks in a humidified 5% CO_2_ environmental incubator at 37 °C. After the cells grow adherently at the bottom of flask to form colonies, it was digested and separated with trypsin for seeding.

#### Cell viability experiments

The in vitro cytotoxicity was carried out by the quantitative MTT assay. Firstly, the films prepared under different electrodeposition conditions (0.36–0.9 C) were cut into 8 mm diameter disks and sterilized by UV light overnight. The films were placed on the bottom of 24 well plate, and then cells were seeded with a density of 10^4^ cells per well. After incubating for a predetermined time, the cell activity of each group was carried out by MTT method. Cell viability was calculated by assuming 100% viability in the control set (media without films).

### In vivo antimicrobial activity of Cu^2+^-Alg films

All animal experiments adhered to the NIH guidelines for the care and use of laboratory animals (NIH Publication no. 85-23 Rev. 1985) and were approved by the Research Center for Laboratory Animals of Shanghai University of Traditional Chinese Medicine. Male Sprague Dawley (SD) rat (180–200 g) were divided into three groups: (i) (Ca^2+^-Alg)_0.36C_; (ii) (Cu^2+^-Alg)_0.36C_; (iii) commercial Ag dressing Biatain^®^. Each group contains eight replicate samples, four samples were evaluated for 1 day and the rest were evaluated for 3 days. Two wounds with 1–2 cm in length were made on the back of each rat. All the sample films with 8 mm in diameter were individually pre-seeded with 10 μL of inoculums containing 10^8^ CFU/mL of MRSA, and then air-dried. After that, each sample film was implanted into one incision. After 1 day and 3 days’ implantation, the animals were euthanized by inhalant anesthetic and the bacteria on wound site were quantified by dilution plate assay. The wounds after 3 days’ treatment were excised and fixed in 10% formaldehyde, then embedded in paraffin, cut into sections, and finally stained with H&E dye.

### Statistical analysis

All data were expressed as mean and standard deviation (SD) and analyzed using one-way ANOVA with post hoc tests. Significance was set at *p* < 0.05 (***p < 0.001, **p < 0.01, *p < 0.05), while *p* > 0.05 was considered to be statistically non-significant.

## Results and discussions

### Characterization of copper alginate films

In this study, we used an anodic deposition strategy to fabricate Cu^2+^-Alg films. Experimentally, Cu_*2*_(OH)_*2*_CO_3_ powders (0.5 wt%) were blended into a sodium alginate (0.8 wt%) solution along with stirring for 5 h. After being fully mixed, a three-electrode system was immersed into the electrolyte to electrodeposit materials using a constant current control for a predetermined time. After a few seconds, a uniform copper alginate hydrogel film formed in the anode surface. A self-supporting film was easily to be obtained by carefully peeling off the materials from the electrode. The corresponding dry film was obtained by extensive rinsing with ultrapure water and then freeze-drying. (Cu^2+^-Alg)q is used to denote the obtained film (q indicates the consumed charge amount).

**Fig. 1:**
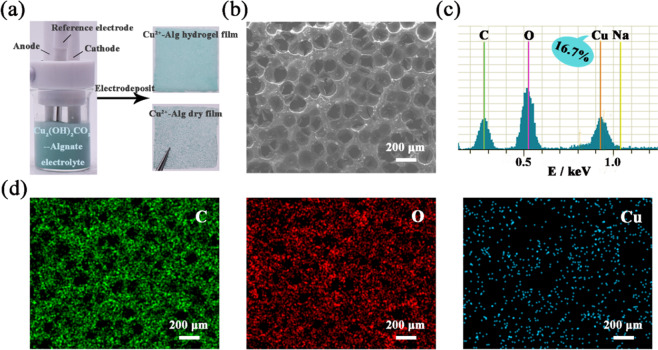
**a** The photographs of gel and dry Cu^2+^-Alg films prepared by electrodeposition. **b** Surface SEM image of (Cu^2+^-Alg)_0.54C_. **c** EDS spectra of (Cu^2+^-Alg)_0.54C_. **d** EDS mapping of C, O, and Cu elements within the dressing, respectively

Figure 1a shows the photographs of Cu^2+^-Alg film prepared by electrodeposition using a constant current density of 2.67 mA/cm^2^ for 90 s. The total consumed charges are summed as 0.54 C. The left indicates the employed three-electrode system immersed with Cu_2_(OH)_2_CO_3_-Alginate electrolyte. The right upper indicates the freshly prepared (Cu^2+^-Alg)_0.54C_ film appearing a uniform light blue, which is due to the incorporation of Cu^2+^. The right lower shows a sponge-like morphology of freeze-dried (Cu^2+^-Alg)_0.54C_ film. The dry film was further used for morphological observation. SEM observation in Fig. 1b reveals many open pores with ~135 μm in diameter are distributed on film surface (Fig. S1 shows the histogram of pore size distribution), which are presumably generated by the released CO_2_ and O_2_ near the anode. The porosity from the interconnected pores was about 51.2%, characterized by a wicking test. The uniform and porous structure might be capable to improve the water uptake and permeability of copper alginate films which are benefit for wound treatment.

The elemental analysis and compositional distribution of the dressings were further investigated by EDS mapping. Figure 1c shows that copper ions were successfully integrated into the (Cu^2+^-Alg)_0.54C_ film, with the content of 16.7%. The images in Fig. 1d are elemental mappings of C, O, and Cu, respectively. The results prove the continuously and uniformly spatial distribution of Cu^2+^ within the dressing. Therefore, electrodeposition is a superior method in preparing uniform copper alginate materials within a short time.

### Continuous modulation of films’ thickness and copper content

**Fig. 2:**
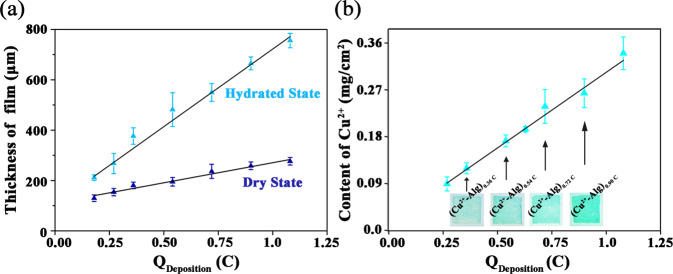
The linear correlation between the film thickness (**a**) and copper contents (**b**) with the consumed charges for Cu^2+^-Alg fabrication

Figure 2a illustrates the correlation between the resultant film thickness and the amount of consumed charges. The thickness of hydrated films was measured by electronic spiral micrometer and the thickness of dry films was measured via SEM imaging of the cross sections of freeze-dried films. It can be seen that with the increase of charges, the deposited film is thicker and it indicates a linear relationship between film thickness and charge transfer both in dry and hydrated state. In principle, the increased anode charges induce more free Cu^2+^ to be generated near the anode, which further cross-link and deposit sodium alginates, and eventually make the hydrogel film thicker.

Figure 2b insert shows photos of the Cu^2+^-Alg hydrogel films electrodeposited under different charge conditions. All the hydrogel films with different charge conditions show the characteristic blue color of Cu^2+^, and the color is getting darker along with the increase of charge. This is a visual evidence to illustrate the relationship between Cu^2+^ and consumed charges.

In order to quantify the exact content of Cu^2+^ ions that were incorporated inside the alginate films, we first disintegrated Cu^2+^-Alg hydrogel film by competitively chelating Cu^2+^ with citrate ions and then performed an ICP-AES measurement to test the content of released Cu^2+^. As indicated by the plot in Fig. 2b, the Cu^2+^ content and consumed charges have a linear relationship. It can be explained by the same mechanism as mentioned above: the amount of electricity consumed during the electrodeposition determines the amount of H^+^ produced by the anode electrolysis, which further determines the Cu^2+^ generated by the reaction of H^+^ with suspended Cu_*2*_(OH)_*2*_CO_3_ particles. Therefore, when the amount of electricity increases, more H^+^ react with Cu_*2*_(OH)_*2*_CO_3_ particles to liberate more Cu^2+^ to construct the Cu^2+^-Alg film.

Taken together, these results reveal that Cu^2+^-Alg films can be continuously and controllably prepared with corresponding physical structure (thickness) and chemical composition (copper content) by changing electrodeposition parameters.

Besides, all above factors will further influence the films’ mechanical properties. Figure S2a–c shows that with the imposed charge increase, the breaking strength of the films also increase, the group (Cu^2+^-Alg)_1.08C_ can reach about 3.94 MPa. While there is no big difference between the breaking tensile strain of different Cu^2+^-Alg films, which all are about 4%.

### In vitro antimicrobial activity of Cu^2+^-Alg films

Cu^2+^ is considered to possess a broad spectrum of antibacterial properties, so we believe that the Cu^2+^-Alg films obtained by electrodeposition also have strong antibacterial activity.

To examine the antimicrobial activities of our films, *S. aureus* and *E. coli* were used in these antimicrobial experiments as gram-positive bacteria model and gram-negative bacteria model, respectively. We placed the film (Cu^2+^-Alg)_0.54C_ in a 24 well plate with bacteria at a concentration of 10^7^ CFU/mL. Then, the antimicrobial activity was systematically evaluated after incubation in a 37 °C bacterial incubator for 0.5 and 10 h. In this study, a commercial Alginate Ag dressing (Biatain^®^) was used as the positive control group and the blank well plate was set as the negative control group. The commercial Alginate Ag dressing (Biatain^®^) is composed of calcium alginate, carboxymethyl cellulose sodium, polyethylene glycol, and silver ion compound (zirconium sodium phosphate containing silver), which can release silver ions to perform the antimicrobial function in the presence of wound exudate when covered to the wound surface [49].

**Fig. 3:**
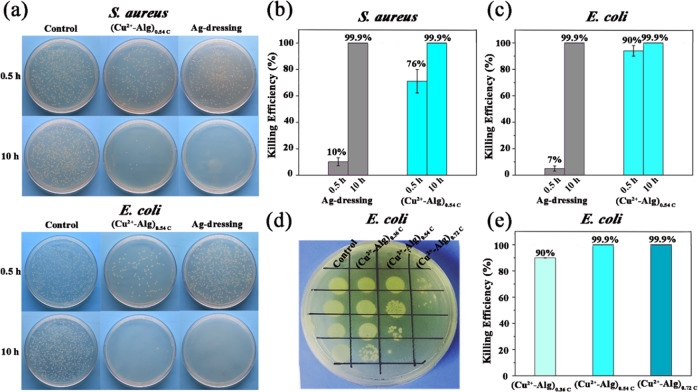
In vitro antimicrobial activities. **a** Photographs of *S. aureus* and *E. coli* colonies on the ager plate after incubation with different samples and gradient dilution. **b** Bacterial killing efficiency of (Cu^2+^-Alg)_0.54C_ and commercial Ag dressing against *S. aureus* and **c** *E. coli*. **d** Photographs of *E. coli* colonies on the ager plate after incubation with Cu^2+^-Alg films prepared by different charge conditions and gradient dilution. **e** Bacterial killing efficiency of electrodeposited Cu^2+^-Alg films prepared by different charge conditions. Bacteria incubated in the blank well plate were set as the control

At first, we performed plate counting to assess bacterial viability as illustrated in Fig. 3a. The upper images show (Cu^2+^-Alg)_0.54C_ performed a lower plate count of *S. aureus*, indicating the strongest antimicrobial activities at 0.5 h. After prolonging the incubation time to 10 h, it can be seen that the plate counts of (Cu^2+^-Alg)_0.54C_ and the Ag dressing were both significantly reduced compared to the blank control. The lower photographs in Fig. 3a reveals that *E. coli* responded similarly to (Cu^2+^-Alg)_0.54C_ film and Ag dressing.

Quantification of these results are presented at the right in Fig. 3b, c. The results show that after incubation for 0.5 h, the (Cu^2+^-Alg)_0.54C_ dressing performed a good antibacterial effect on both bacteria, with the ratio of 76% and 90% respectively, while the killing efficiency of Ag dressing were less than 10%. After the incubation time was extended to 10 h, both (Cu^2+^-Alg)_0.54C_ film and Ag-dressing’s bactericidal rate reached 99.9%, exhibiting comparable antibacterial abilities. This result illustrates that the (Cu^2+^-Alg)_0.54C_ film has a faster working time than commercial Ag dressing, which is essential for preventing the infected wound from deteriorating [50]. It might be ascribed to the faster-releasing speed of copper ions comparing to silver elements from their corresponsive polymer matrixes, which results in a distinct cell response at the early stage.

We further compared the antibacterial effect of Cu^2+^-Alg films prepared under different conditions. Three groups (Cu^2+^-Alg)_0.36C_, (Cu^2+^-Alg)_0.54C_, (Cu^2+^-Alg)_0.72C_ were used to incubate with *E. coli* for 10 h, the results are shown in Fig. 3d, e. It can be seen that (Cu^2+^-Alg)_0.54C_ and (Cu^2+^-Alg)_0.72C_ showed an extremely high bactericidal rate of 99.9% against *E. coli*, while the killing efficiency of (Cu^2+^-Alg)_0.36C_ reached to 90%, which was lower than the former two groups. This can be explained by the relatively lowered Cu^2+^ content in (Cu^2+^-Alg)_0.36C_ (referring to Fig. 2b).

### Antibacterial mechanism

**Fig. 4:**
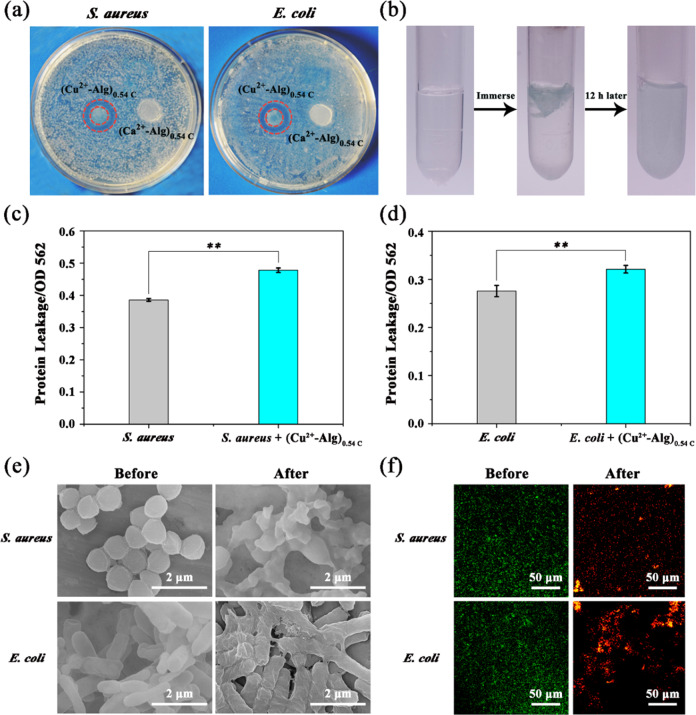
**a** (Cu^2+^-Alg)_0.54C_ film generates a zone of bacteria inhibition in an agar plate assay. **b** The decomposing process of (Cu^2+^-Alg)_0.54C_ film in PBS buffer. **c** Protein leakage of and *S. aureus*. **d** *E. coli* after incubation with (Cu^2+^-Alg)_0.54C_ film for 10 h. The higher value of OD 562 indicates higher protein leakage, which originates from cell membrane damage. **e** SEM images of *E. coli* and *S. aureus* before and after film treatment. Scale bar is 2 μm. **f** Live and dead staining images of *E. coli* and *S. aureus* before and after film treatment. Green color indicates live and dead bacteria; red color indicates dead bacteria. Scale bar is 50 μm

Next, we performed several studies to reveal the antibacterial mechanism of the Cu^2+^-Alg films. An inhibition zone assay was initiated to determine the contribution of Cu^2+^ for bacterial inhibition. In these experiments, trimmed film samples were placed onto the surface of an agar plate to make it in close contact with bacteria, and incubated at 37 °C for 12 h. Here we use the electrodeposited (Ca^2+^-Alg)_0.54C_ film reported in our previous work as the negative control group. The results in Fig. 4a indicate that after contact with *S. aureus* and *E. coli*, (Cu^2+^-Alg)_0.54C_ created an obvious growth inhibition-zone, which was attributed to the release of diffusible Cu^2+^. No inhibition-zone was observed for the control films ((Ca^2+^-Alg)_0.54C_). We then examined the (Cu^2+^-Alg)_0.54C_ film’s decomposing in PBS buffer, as shown in Fig. 4b. It can be seen that the film collapsed completely after 12 h soaking and the solution became light blue, demonstrating a Cu^2+^ diffusion determined antibacterial activity. Mechanistically, it is believed that Cu^2+^ kills bacteria firstly through damage or destruction of the cell membrane [51–53], and such damage is assessed by measuring protein leakage associated with such permeability changes [54, 55]. According to Fig. 4c, d, after incubation with (Cu^2+^-Alg)_0.54C_ film for 10 h, *S. aureus* and *E. coli* exhibited statistically higher protein leakage compared to the control group, demonstrating that the bacterial membrane has experienced a severe damage.

We then examined if incubation with (Cu^2+^-Alg)_0.54C_ film induced significant morphological changes to bacteria. The SEM images in Fig. 4e show that the original shape of *S. aureus* was spherical, and *E. coli* had a rod-like shape, both in a normal growth state. After the treatment with (Cu^2+^-Alg)_0.54C_ films, the morphology of the bacteria changed greatly, with disruption of the cellular structure and the loss of original appearance. This result further confirms the conclusion that Cu^2+^ can cause membrane disruption and structural deformation of bacteria.

Finally, a live (green)/dead (red) staining assay was applied in visual observation of bacterial state. In this experiment, the bacteria were incubated for 10 h with (Cu^2+^-Alg)_0.54C_, before and after which they were stained for viability using the fluorescence dyes SYTO 9 and PI. The confocal images in Fig. 4f show a large number of green (viable) bacteria before film treatment, while a large number of yellow or red bacteria were observed after incubation with (Cu^2+^-Alg)_0.54C_ films, demonstrating that both bacteria have almost died [56].

In summary, the results in both Fig. 3 and Fig. 4 exhibit the great antibacterial effect of electrodeposited Cu^2+^-Alg films both against gram-positive and gram-negative bacteria through releasing Cu^2+^ to destruct the cell membrane of bacteria.

### Cell viability of Cu^2+^-Alg films

Biocompatibility is one of the basic requirements for biomedical materials for wound treatment. To study the cytotoxicity of prepared Cu^2+^-Alg films, HUVECs were co-cultured with different Cu^2+^-Alg films for 24 h. An MTT assay was performed to evaluate the cell viability.

**Fig. 5:**
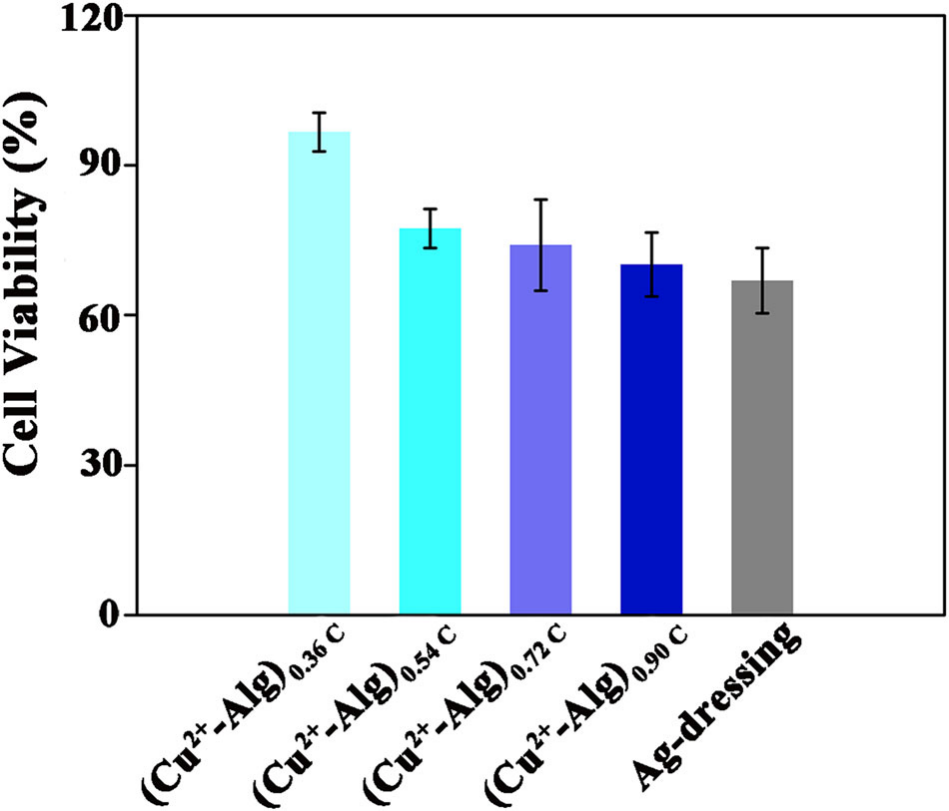
The biocompatibility estimation of films using a MTT viability assay. HUVECs cells were cultured in the presence of different films for 1 day. HUVECs cell viability assays provide evidence that all the Cu^2+^-Alg films have comparable or better cell compatibility than commercial Ag dressing

Figure 5 indicated that the viabilities of cells treated with (Cu^2+^-Alg)_0.36C_, (Cu^2+^-Alg)_0.54C_, (Cu^2+^-Alg)_0.72C_, (Cu^2+^-Alg)_0.90C_, and Ag-dressing were: 97, 77, 74, 70, 68%, respectively. And all of these Cu^2+^-Alg films behaved comparable or better cell compatibility than commercial Ag dressing. Varying consumed charges can effectively modulate the biocompatibility of Cu^2+^-Alg films. Remarkably, the electrodeposited Cu^2+^-Alg films can be adjusted to possess both high antimicrobial capability and good biocompatibility just by charge transfer (Q).

### In vivo antimicrobial activity of Cu^2+^-Alg films

To evaluate the in vivo antimicrobial activity, we used an infected SD rat model in which bacteria contaminated films were implanted subcutaneously. MRSA was employed as a model bacterium for this study since it is responsible for several difficult-to-treat infections in humans. Three groups were set up in the experiment: (Ca^2+^-Alg)_0.36C_, (Cu^2+^-Alg)_0.36C_, and commercial silver dressing. Two 1–2 cm length incisions were created in the dorsum of each rat, then the films were implanted into the wound site after seeding 10 μL of MRSA (10^8^ CFU/mL). All groups were sutured after material implantation.

**Fig. 6:**
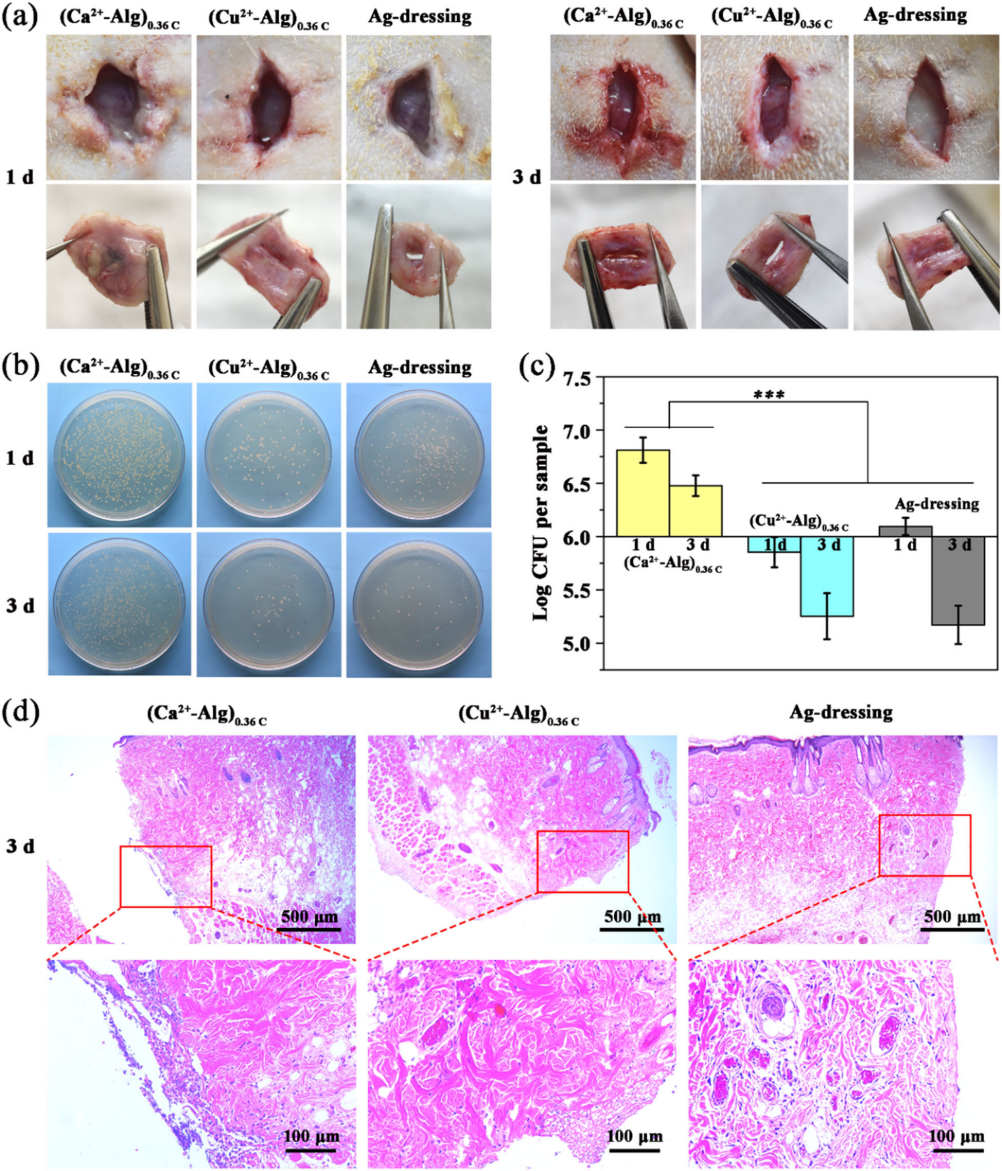
In vivo antimicrobial studies by employing an infected SD rat model. **a** Photographs of re-opened incisions with different treatments after 1 day and 3 days. **b** Exudate bacterial plate counting and **c** quantitative summary plot at day 1 and day 3 postoperatively. **d** Histological analysis of implantation sites after 3 days’ treatment. Scale bar is 500 and 100 μm, respectively

1 day and 3 days after surgery, the animals were sacrificed and wounds were reopened. The infection state was photographed as shown in Fig. 6a. It can be seen that after 1 day and 3 days, the wounds implanted with the (Cu^2+^-Alg)_0.36C_ film and Ag dressing presented a slight suppuration, while the control wound treated with (Ca^2+^-Alg)_0.36C_ had obvious inflammation and serious suppuration, indicating that the wound was severely infected.

Each wound site was wiped with cotton swabs to collect bacteria, and diluted plate counts were performed to evaluate the antibacterial efficiency (Fig. 6b). The results in Fig. 6c illustrate that after 1 day and 3 days’ implantation, the considerably lower bacterial colonies were observed in the incision implanted with (Cu^2+^-Alg)_0.36C_, verifying a successful inhibition on the microbe growth that can be comparable with commercial silver dressing. Statistically, we observed an average over 1-Log reduction of bacterial number in the implantation site of (Cu^2+^-Alg)_0.36C_ in comparison with the (Ca^2+^-Alg)_0.36C_ group.

Histological evaluation of the adjacent skin tissue after 3 days’ film implantation was also carried out by hematoxylin and eosin stained sections (H&E staining). In Fig. 6d, it is obvious that a large number of inflammatory cells existed in the control groups ((Ca^2+^-Alg)_0.36C_) implying that the possible infection and immune response occurred. Instead, a significant decrease of inflammatory cells is observed in the (Cu^2+^-Alg)_0.36C_ and Ag-dressing groups. These results verified the strong antibacterial capability and good tissue compatibility of copper alginate films in vivo.

## Conclusions

In summary, we report a functional antibacterial Cu^2+^-Alg dressing prepared by a novel electro-fabrication methodology, which is much more convenient, efficient, and controllable compared to the traditional strategies. The generated film possesses a porous structure and excellent uniformity. More importantly, the Cu^2+^ content and thickness of the film can be continuously regulated by adjusting the electricity consumption during fabrication. At the same time, the Cu^2+^-Alg films have strong antimicrobial activities both in vitro and in vivo through releasing Cu^2+^ to destroy the cell membrane of bacteria, which was equivalent to commercial Ag dressings, but has a shorter onset time. The cytotoxicity and antibacterial efficiency of Cu^2+^-Alg dressings were both positively related to Cu^2+^ content. Therefore, the controllability of electro-fabrication makes it possible to endow Cu^2+^-Alg dressings with both high antimicrobial capability and good biocompatibility just by adjusting charge transfer.

In view of the controllability, uniformity and excellent antibacterial properties of the electrodeposited Cu^2+^-Alg film, we envision this film has great potential value in the field of antibacterial dressings.

## Supplementary information

Supplementary Information
