# Supplementary material for: Continuous and controllable electro-fabrication of antimicrobial copper-alginate dressing for infected wounds treatment
Source: J Mater Sci Mater Med. 2021 Nov 24;32(12):143. doi: 10.1007/s10856-021-06619-2 (PMC8613166; doi:10.1007/s10856-021-06619-2)
Supplement: Supplementary file 1 — Supplementary Information [file 10856_2021_6619_MOESM1_ESM.docx]

Continuous and Controllable Electro-fabrication of Antimicrobial Copper-Alginate Dressing for Infected Wounds Treatment

Shijia Wang ^1,†^, Xiaoli Liu ^1,†^, Miao Lei ^1^, Junjie Sun ^1^, Xue Qu ^1,^* and Changsheng Liu ^1,^*

^1^ Key Laboratory for Ultrafine Materials of Ministry of Education, Frontiers Science Center for Materiobiology and Dynamic Chemistry, School of material science and engineering, East China University of Science and Technology, Shanghai 200237, China

***** Correspondence: quxue@ecust.edu.cn (Xue Qu); liucs@ecust.edu.cn (Changsheng Liu)

**^†^** These authors contributed equally to this work


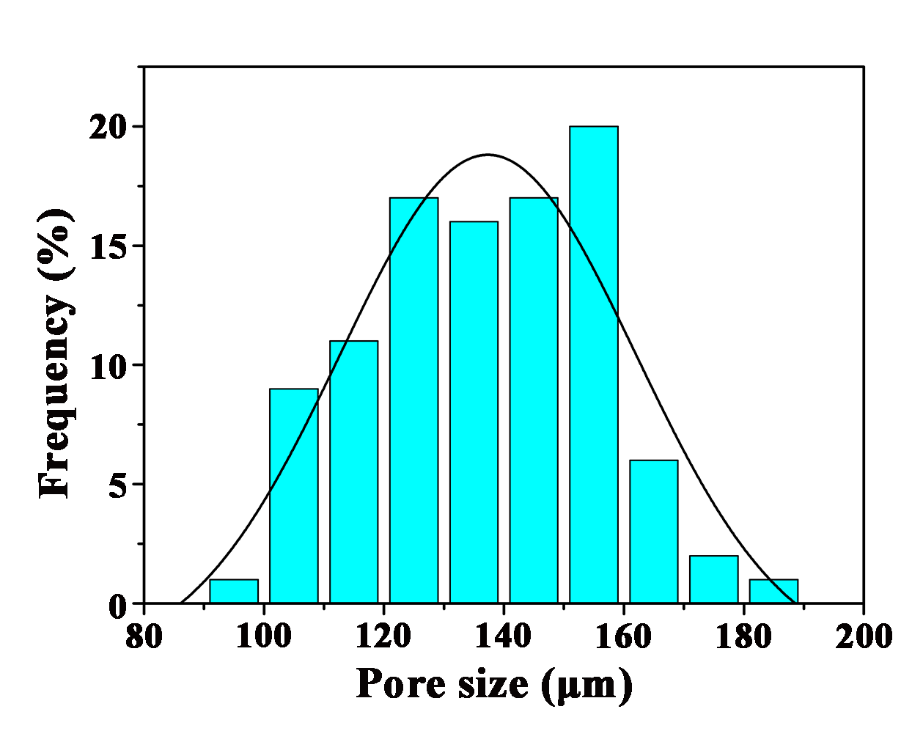


**Fig. S1.** Histogram of pore size distribution of the film (Cu^2+^-Alg)_0.54C_ (n=100).

**Table. S1.** The pore connectivity of the film (Cu^2+^-Alg)_0.54C_ evaluated by an ethanol-wicking technique (n=5).

| **Sample No.** | **1** | **2** | **3** | **4** | **5** | **Average** |
| --- | --- | --- | --- | --- | --- | --- |
| Interconnected void volume / mm^3^ | 8.872 | 10.773 | 10.520 | 8.619 | 10.139 | / |
| Total volume / mm^3^ | 17.800 | 19.959 | 20.475 | 17.145 | 20.140 | / |
| Interconnected porosity / % | 49.8 | 54.0 | 51.4 | 50.3 | 50.3 | 51.2 ± 1.7 |


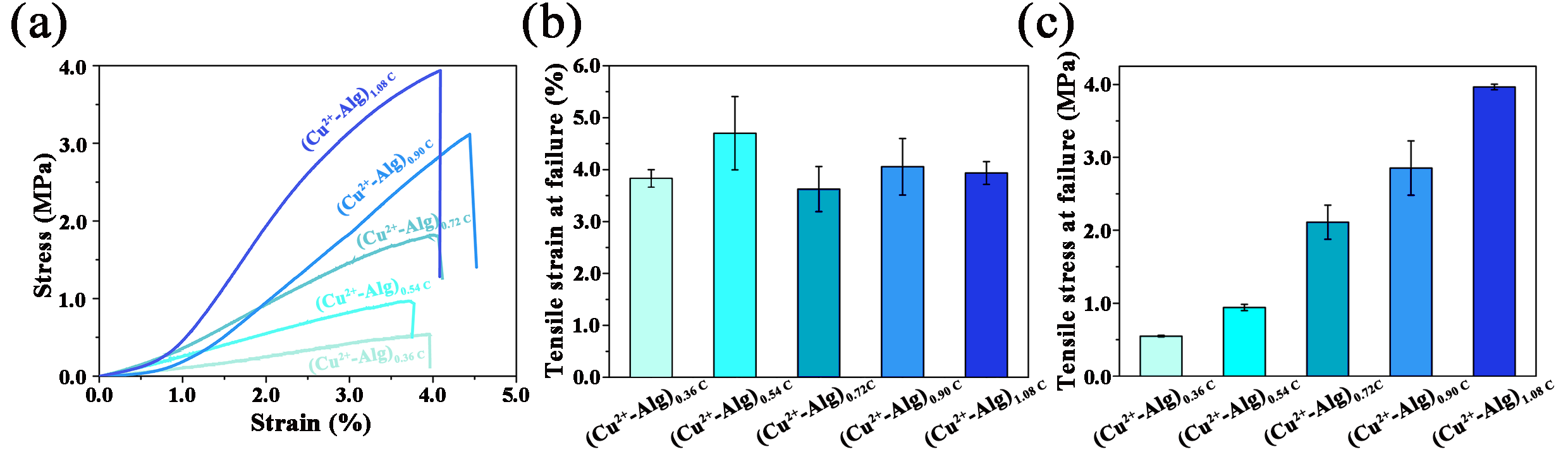


**Fig. S2.** The tensile stress–strain curves of Cu^2+^-Alg films.
